# Evaluation of Drug-Associated Phototoxicity Under 980 nm Near-Infrared Laser Irradiation in Human Dermal Fibroblasts

**DOI:** 10.3390/pharmaceutics18101259

**Published:** 2026-10-06

**Authors:** Wiktoria Odrzywołek, Zuzanna Rzepka, Małgorzata Bożek, Artur Beberok, Dorota Wrześniok, Sławomir Wilczyński

**Affiliations:** 1 Department of Basic Biomedical Science, Faculty of Pharmaceutical Sciences in Sosnowiec, Medical University of Silesia, Jedności 8b, 41-200 Sosnowiec, Poland; wiktoria.odrzywolek@sum.edu.pl (W.O.); malgorzata.bozek@sum.edu.pl (M.B.); 2 Department of Pharmaceutical Chemistry, Faculty of Pharmaceutical Sciences in Sosnowiec, Medical University of Silesia, 4 Jagiellońska Str., 41-200 Sosnowiec, Poland; zrzepka@sum.edu.pl (Z.R.); abeberok@sum.edu.pl (A.B.); dwrzesniok@sum.edu.pl (D.W.)

**Keywords:** drug-induced photosensitivity, phototoxicity, near-infrared laser

## Abstract

**Background:** Many widely used drugs—including non-steroidal anti-inflammatory drugs, tetracyclines and thiazide diuretics—are labelled as photosensitising, yet their phototoxicity has been defined almost entirely by ultraviolet A (UVA) exposure. Whether this risk extends to the near-infrared (NIR) wavelengths of lasers commonly used in aesthetic medicine is unclear, and a history of such medication is often treated as a relative contraindication to laser procedures. **Methods:** A bibliometric analysis of 391 Scopus-indexed publications using VOSviewer was performed to quantitatively map the thematic structure of the retrieved literature. The bibliometric mapping was complemented by sequential refinement of the Scopus corpus and a focused assessment of the final records to determine whether studies directly addressing the predefined experimental question could be identified. We assessed whether 980 nm diode-laser irradiation of normal human dermal fibroblasts (HDFs) induces phototoxicity in the presence of seven photosensitisers (ketoprofen, meloxicam, hydrochlorothiazide, doxycycline and diclofenac, with chlorpromazine and 8-methoxypsoralen as reference photosensitizers). Cells were irradiated through the closed culture-plate lid at two fluences (62.3 and 34.4 mJ/cm and viability was measured 0.5, 1 and 24 h later. Data were analysed by two-way ANOVA (compound × laser), pairwise Welch tests with Benjamini–Hochberg correction, and equivalence testing against an ISO 10993-5-based margin. **Results:** Viability remained between 82.7% and 109.6% of control under all conditions and never fell below the 70% cytotoxicity threshold. The compound × laser interaction was non-significant at every time point (*p* = 0.25–0.69), indicating no drug-specific phototoxicity, and no pairwise comparison remained significant after correction. A small, drug- and fluence-independent reduction of about six percentage points was observed but stayed well within the non-cytotoxic range and was equally present in drug-free controls. **Conclusions:** Under the specific 980 nm irradiation conditions tested, the photosensitising drugs did not increase phototoxicity in normal human dermal fibroblasts. These findings support a wavelength-specific view of drug photosafety: rigorous UVA-directed photoprotection remains essential, whereas the tested drugs did not increase phototoxicity under the specific in vitro 980 nm irradiation conditions evaluated in this study.

## 1. Introduction

Drug-induced photosensitivity is one of the most frequent cutaneous adverse reactions to systemically and topically administered medicines, and its reported incidence has grown in parallel with the widespread use of photoreactive drugs and with increasing recreational and occupational sun exposure [1,2]. Two mechanistically distinct entities are recognised. Phototoxic reactions are non-immunological, dose-dependent and may occur in any individual on first exposure, clinically resembling an exaggerated sunburn confined to light-exposed skin. Photoallergic reactions, by contrast, are comparatively rare, immunologically mediated, delayed and eczematous, and require prior sensitisation [2,3,4]. Because phototoxicity is by far the more common of the two and can be reproduced in cultured cells, it is the reaction of principal concern whenever the photosafety of a drug is assessed [3,5].

At the molecular level, both reactions begin with the absorption of radiant energy by a chromophore—the parent drug or one of its photoproducts [1,4]. Absorption promotes the molecule to an electronically excited singlet and then triplet state, from which two principal photochemical routes ensue: type I reactions generate radical species by electron or hydrogen transfer, whereas type II reactions transfer energy to molecular oxygen to yield singlet oxygen and other reactive oxygen species. The resulting oxidative burden damages membrane lipids, proteins and nuclear DNA and can trigger mitochondrial dysfunction and apoptosis [1,6]. A critical and often underappreciated corollary of this mechanism is that a reaction can only proceed at wavelengths that the chromophore actually absorbs. For the overwhelming majority of photosensitising drugs, the relevant absorption—and therefore the clinical action spectrum—lies within the ultraviolet A (UVA, 315–400 nm) region, with smaller contributions from ultraviolet B (UVB, 280–315 nm) and, only exceptionally, visible light [2,3,4].

The compounds examined in the present study belong to several therapeutic classes, but share a common feature: their photoreactivity is predominantly associated with ultraviolet radiation. Among the non-steroidal anti-inflammatory drugs, ketoprofen is one of the most frequently reported photosensitisers; its benzophenone chromophore absorbs in the UVA range and, upon irradiation, potentiates cell death through reactive oxygen species generation, lipid peroxidation and DNA damage [6,7]. Diclofenac and the oxicam derivative meloxicam behave analogously, producing UV-dependent oxidative injury in keratinocytes, melanocytes and dermal fibroblasts [8,9,10]. The tetracycline antibiotic doxycycline is the most phototoxic member of its class, with an action spectrum dominated by long-wave UVA [11,12]. The thiazide diuretic hydrochlorothiazide is a well-recognised photosensitiser that enhances the cutaneous response to ultraviolet radiation and has been the subject of pharmacovigilance and controlled photobiological investigation because of its possible photocarcinogenic potential [13]. Finally, the phenothiazine chlorpromazine and the furocoumarin 8-methoxypsoralen are classical, well-characterised photosensitisers that act only after UVA activation and are used internationally as reference (positive-control) substances in validated phototoxicity assays [1,14,15]. In every one of these cases, the photoreactive window is confined to the ultraviolet range.

This mechanistic understanding is embodied in the standard regulatory test for photoreactivity, the in vitro 3T3 Neutral Red Uptake phototoxicity assay (OECD Test Guideline 432) in which cells are exposed to a substance in the presence and absence of a non-cytotoxic dose of UVA [15]. The guideline restricts the light source to the UVA/visible region and considers a compound unlikely to be photoreactive when its molar extinction coefficient is negligible at those wavelengths [15]. The entire body of evidence that underpins the “photosensitising” label attached to the drugs above therefore rests on ultraviolet, and predominantly UVA, exposure.

In parallel, lasers emitting in the red and near-infrared region have become ubiquitous in medicine. The 980 nm indium–gallium–arsenide diode laser in particular is now routinely employed in oral and maxillofacial surgery, dermatology, phlebology, laser-assisted lipolysis and aesthetic medicine, where it is valued for its ability to induce haemostasis, its selective absorption by water and haemoglobin, and its comparatively deep tissue penetration [16,17]. Near-infrared photons penetrate the dermis far more efficiently than ultraviolet radiation and deliver their energy to resident cells such as fibroblasts rather than being absorbed superficially [18]. At lower fluences, the same wavelength is exploited therapeutically for photobiomodulation, modulating fibroblast proliferation, mitochondrial activity and wound healing [19,20,21]. Crucially, 980 nm lies far outside the ultraviolet absorption bands of the photosensitising drugs discussed above, so the fundamental prerequisite for a classical phototoxic reaction—chromophore absorption of the incident light—is not obviously met [18,19].

Despite this spectral mismatch, the phototoxic potential attributed to these drugs is frequently generalised in clinical practice to all forms of light exposure, including laser- and near-infrared-based procedures, largely by analogy and in the absence of direct experimental evidence. This issue is of particular importance in aesthetic medicine, where laser-based treatments are performed on a very large scale and where a reported history of photosensitising medication—including the non-steroidal anti-inflammatory drugs examined here and other agents—is frequently regarded as a relative contraindication, prompting clinicians to postpone or withhold laser procedures. Whether such caution is warranted for near-infrared laser exposure remains unclear. From a photochemical perspective, the relevant question is whether photosensitising drugs retain phototoxic potential at 980 nm, where their ultraviolet chromophores are not efficiently excited. Whether a drug that is unequivocally phototoxic under UVA retains any phototoxicity when the activating light is a longer-wavelength laser remains, from a photochemical standpoint, an open and testable question: if chromophore absorption is the rate-limiting step, no phototoxic response should be expected at 980 nm. Data addressing this question in normal, non-malignant human skin cells are scarce.

The present study was designed to address this gap. Using normal human dermal fibroblasts as a physiologically relevant model of the dermal compartment reached by near-infrared light, we evaluated whether irradiation with a 980 nm diode laser, delivered at two low energy densities (62.3 and 34.4 mJ/cm^2^, corresponding to source-to-plate distances of 1.5 and 2.5 cm) modifies cell viability in the presence of seven established photosensitising drugs—ketoprofen, meloxicam, hydrochlorothiazide, doxycycline and diclofenac, together with chlorpromazine and 8-methoxypsoralen as reference photosensitisers—each tested at two concentrations and assessed 0.5, 1 and 24 h after exposure. We hypothesised that, in contrast to the well-documented UVA-driven phototoxicity of these agents, near-infrared laser irradiation would not induce a comparable loss of fibroblast viability, and that the routine extrapolation of ultraviolet-based phototoxicity profiles to laser wavelengths therefore warrants critical re-examination.

## 2. Materials and Methods

### 2.1. Bibliometric Analysis and Research Gap Identification

#### 2.1.1. Database Search Strategy and Selection Criteria

To assess the volume of existing literature and delineate the research gap regarding NIR-associated phototoxicity, the Scopus database was searched on 22 July 2026 using the following query: TITLE-ABS-KEY ((photosensitiz* OR phototoxici*) AND (“near infrared” OR “980 nm” OR “diode laser”) AND NOT (“photodynamic therapy” OR PDT OR cancer)). The exclusion component was intended to reduce the contribution of conventional photodynamic therapy, particularly oncology-related literature, and to focus the retrieved corpus on drug-related phototoxicity in the context of NIR and diode-laser exposure, rather than to exclude all photodynamic phenomena. The search results were limited to English-language articles and reviews within Medicine; Pharmacology, Toxicology and Pharmaceutics; and Biochemistry, Genetics and Molecular Biology. After applying the search strategy and these limits, 391 records were retained as the bibliometric corpus. A two-step database refinement was performed using the Scopus “Search within results” function. In the first step, the query fibroblast OR dermal OR “aesthetic medicine” OR ketoprofen OR meloxicam OR hydrochlorothiazide OR doxycycline OR diclofenac reduced the initial corpus from 391 to 49 records. In the second step, the 49 records were further refined using ketoprofen OR meloxicam OR hydrochlorothiazide OR doxycycline OR diclofenac, resulting in 9 records. These database-based refinement steps did not constitute manual title/abstract screening. The nine records obtained after the final refinement step were subsequently catalogued and assessed by the authors for their direct relevance to the research question.

#### 2.1.2. Conceptual Mapping in VOSviewer

For the selected dataset of 391 publications, an author keyword co-occurrence analysis was performed using VOSviewer software (1.6.21). To ensure methodological rigor and eliminate lexical noise, a customized thesaurus file was implemented. This allowed for the consolidation of synonyms and spelling variants (such as unifying “near infrared” with “near-infrared” and standardizing terms related to photodynamic inactivation) while filtering out purely methodological or computational descriptors (e.g., “dft”). Setting the minimum keyword occurrence threshold to 4 yielded 25 core conceptual nodes from an initial total of 1017 unique author-defined keywords.

### 2.2. Reagents and Test Compounds

Seven photosensitising drugs were investigated: five test drugs—ketoprofen, meloxicam, hydrochlorothiazide, doxycycline and diclofenac—together with two reference photosensitisers, chlorpromazine and 8-methoxypsoralen (all these compounds were obtained from Sigma-Aldrich Inc. Taufkirchen, Germany). Stock solutions were prepared in DMSO and diluted in culture medium immediately before use, so that the final concentration of the vehicle did not exceed 1%. Each drug was applied at two concentrations (C1 (therapeutic): 5 µg/mL for ketoprofen as well as 8-methoxypsoralen, 1.5 µg/mL for meloxicam, 2 µg/mL for doxycycline, 1 µg/mL for diclofenac as well as chlorpromazine; C2:two-fold higher than C1), selected to approximate therapeutically relevant or pharmacologically active exposure levels reported in the literature for each compound and were consistent with concentrations commonly employed in in vitro phototoxicity studies [22,23,24,25,26].

### 2.3. Cell Culture

Normal human dermal fibroblasts (HDFs) (Sigma Aldrich Inc., St. Louis, MO, USA) were used as a physiologically relevant model of the dermal compartment reached by near-infrared light. Cells were maintained in all-in-one fibroblast growth medium (Sigma-Aldrich Inc., Taufkirchen, Germany) at 37 °C in a humidified atmosphere containing 5% CO2. Fibroblasts were cultured on optically clear, tissue-culture-treated polystyrene—the standard substrate for adherent mammalian cells, valued for its optical transparency, biocompatibility and surface treatment that promotes cell attachment. For the experiments, cells were seeded into flat-bottomed, round-well tissue-culture polystyrene 96-well plates fitted with their original transparent polystyrene lids, at a density of 5 × 10^3^ per well, and were allowed to adhere for 48 h to form a sub-confluent monolayer before treatment. The cells used in the in vitro studies were from actual passages 5–9 (counted from the initial isolation of the cells by the supplier).

### 2.4. Experimental Design and Plate Layout

Experiments were performed in 96-well plates according to a fixed layout. The two outermost columns (columns 1 and 12) contained untreated cells and served as the reference control, to which viability was normalised (100%). The five test drugs occupied the intervening columns in adjacent pairs, one pair per drug, each pair corresponding to the two tested concentrations: ketoprofen (columns 2–3), meloxicam (columns 4–5), hydrochlorothiazide (columns 6–7), doxycycline (columns 8–9) and diclofenac (columns 10–11). Within each plate, the upper rows (A–C) were irradiated at a source-to-plate distance of 1.5 cm and the lower rows (D–F) at 2.5 cm, giving three replicate wells per condition. Chlorpromazine and 8-methoxypsoralen were applied in dedicated wells (rows G and H) as positive photosensitising controls.

For each drug, concentration and irradiation distance, the following conditions were compared: (i) untreated control; (ii) drug alone, without irradiation (dark-toxicity control); (iii) laser irradiation without drug; and (iv) drug combined with laser irradiation. A matched set of plates, prepared and handled identically but kept in the dark, was processed in parallel as the non-irradiated (“no-laser”) control. For each combination of drug, concentration and irradiation distance, measurements were performed in six replicate wells.

### 2.5. Laser Irradiation Procedure

Irradiation was performed with a Leonardo^®^ Dual (Biolitec AG, Jena, Germany) diode laser emitting at a wavelength of 980 nm (near-infrared region). The energy delivered to the sample was controlled by adjusting the distance between the laser source and the culture plate. Prior to the main experiments, preliminary dose-finding studies were performed using different irradiation conditions to identify exposure parameters with minimal intrinsic cytotoxicity. Based on these experiments, two working distances were selected: 1.5 cm and 2.5 cm, corresponding to radiant exposures of 62.3 mJ/cm^2^ and 34.4 mJ/cm. Irradiation at a distance of 1.5 cm resulted in an approximately 8% reduction in fibroblast viability, whereas irradiation at 2.5 cm had no detectable effect on cell viability. The laser was operated in pulsed mode at an output power of 2 W with a pulse duration of 0.01 s (10 ms). Each well was exposed to a single laser pulse.

The selection of these conditions was guided by the principle that irradiation used for phototoxicity assessment should itself produce minimal cytotoxicity, thereby allowing a potential drug-dependent enhancement of the irradiation response to be distinguished from direct laser-induced effects. This approach is consistent with the general principle of OECD TG 432, which uses a non-cytotoxic irradiation dose and requires adequate viability of irradiated controls. Because the present study employed human dermal fibroblasts and a 980 nm laser rather than the standard BALB/c 3T3/UVA system, the irradiation conditions were established experimentally.

To preserve the sterility of the cultures, the cell monolayers were irradiated through the closed plate lid; the transparent polystyrene lid was not removed at any stage of the procedure, because lifting it would have exposed the wells to a risk of microbial contamination of the cultures. As the radiation was delivered through the lid, its optical transmittance at 980 nm was determined beforehand: the lid attenuated approximately 10% of the incident radiation (transmittance ≈ 90%). This attenuation was incorporated into all dosimetric calculations, so that the fluences reported below correspond to the radiant exposure actually reaching the cell monolayer. The depth of the culture wells (0.11 cm) and the overlying medium were taken into account in the exposure geometry, and the beam was directed perpendicularly onto the plate. Immediately after irradiation, the plates were returned to the incubator. Furthermore, all experimental results were normalized and interpreted relative to the appropriate untreated control groups, ensuring that the reported effects reflect changes induced by the treatment compared with the corresponding controls.

### 2.6. Cell Viability Assay

Cell viability was assessed at three time points after irradiation—0.5, 1 and 24 h—in both the irradiated plates and the matched non-irradiated (dark) plates, using the WST-1 colorimetric assay. Absorbance was measured at 440 nm and 650 nm (reference wavelength) with a microplate reader (Infinite 200 PRO, TECAN, Männedorf, Switzerland). Viability was expressed as a percentage of the untreated control (defined as 100%), and the results are reported as mean ± standard deviation (SD). For each experimental condition, five technical replicates were performed using individual wells of a 96-well plate. The experiment was independently repeated three times over three consecutive weeks, with each independent run comprising cell seeding, cell growth to the required confluence, drug treatment with or without laser irradiation, and subsequent WST-1 analysis. Thus, the experimental design comprised three independent biological experiments (n = 3), with five technical replicate wells per condition in each experiment. The five technical replicate measurements were averaged within each independent experiment before statistical analysis; therefore, technical replicates were not treated as independent observations.

### 2.7. Photophysical Stability Assessed by UV–Vis Spectrophotometry

To confirm that the applied radiation dose did not alter the chemical structure of the tested drugs, UV-VIS spectroscopy was used to compare ketoprofen before and after 980 nm laser irradiation under the conditions described in Section 2.4. The stability of the investigated drug was evaluated through comparison of its absorption spectra recorded before and after laser irradiation. Absorption spectra were acquired over the UV–Vis range, with the characteristic absorption maximum (λ_max) observed at 260 nm. The spectra of non-irradiated (control) and laser-irradiated samples were compared to identify potential changes in the absorption profile, including shifts in λ_max, variations in absorbance intensity, or the appearance of additional absorption bands that could indicate photochemical degradation or structural modifications. The absence of significant spectral changes following laser exposure was interpreted as evidence of photostability under the applied irradiation conditions.

### 2.8. Statistical Analysis

Cell viability, expressed as a percentage of the untreated control, was analysed using parametric methods after verification of the underlying assumptions. Three independent biological replicates (n = 3) were performed for each experimental condition. No formal a priori sample-size or statistical power calculation was performed, as the study was designed as an exploratory in vitro investigation aimed primarily at screening for potentially harmful effects of the tested treatments under controlled experimental conditions. The sample size was selected based on the experimental design and the need to assess multiple compounds, concentrations, laser conditions, and post-irradiation time points in independent experiments.

Because the primary aim was to assess whether the tested treatments were associated with a biologically relevant reduction in cell viability, a non-significant difference test alone was not considered sufficient to support the absence of a harmful effect. Therefore, the principal analysis was based on equivalence testing using the two one-sided tests (TOST) procedure against a pre-specified equivalence margin of ±20 percentage points. This margin was defined a priori as a practically relevant difference in viability. In parallel, the ISO 10993-5 criterion [22], according to which a reduction in viability below 70% of the untreated control denotes cytotoxicity, was used as a predefined biological benchmark. Treatments were considered statistically equivalent to the untreated control when the 90% confidence interval of the difference was entirely contained within the ±20 percentage-point equivalence margin. Given the exploratory nature of the study and the relatively small number of independent biological replicates, the equivalence analysis was interpreted together with the magnitude of the observed effects, the corresponding confidence intervals, and the predefined ISO 10993-5 cytotoxicity threshold rather than as a substitute for a formal power calculation.

As supporting analyses, a two-way analysis of variance (compound × laser condition) was performed for each time point, with the compound × laser interaction taken as the key test of drug-specific phototoxicity, together with pairwise Welch t-tests of each irradiated condition against its matched non-irradiated counterpart. Correction for multiple comparisons used the Benjamini–Hochberg procedure. A value of *p* < 0.05 was considered significant.

## 3. Results

### 3.1. Results of the Bibliometric Analysis and Quantitative Assessment of the Research Gap

**Figure 1:**
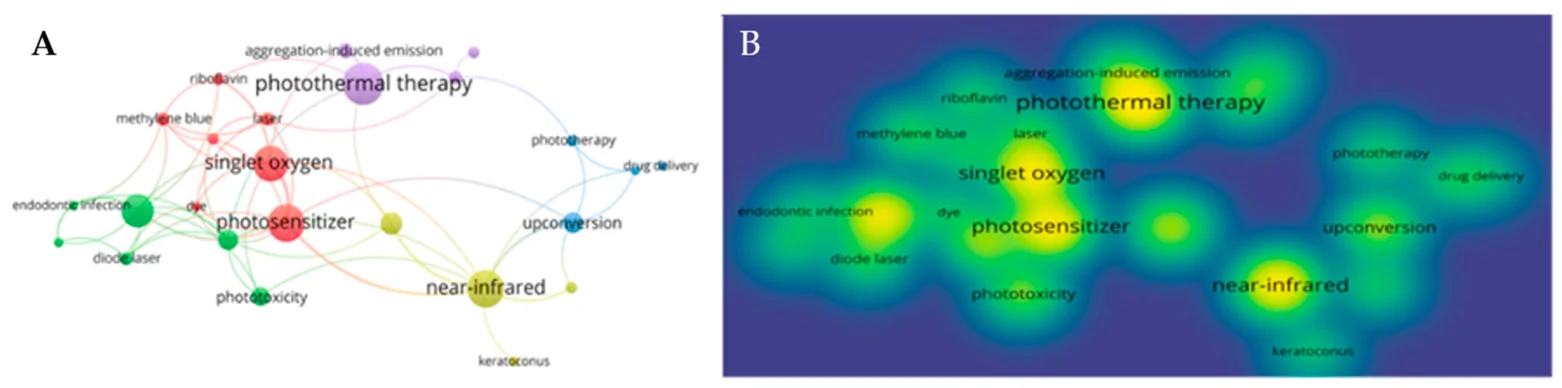
Bibliometric mapping of author keywords from 391 Scopus-indexed publications on laser modalities and phototoxicity (VOSviewer, v.1.6.21). (**A**) Co-occurrence network visualization grouped into five main thematic clusters, where node size reflects keyword frequency and line thickness indicates link strength (**B**) Item density visualization highlighting the principal areas of research concentration within the mapped author-keyword network. Neither the network visualization (**A**) nor the item density map (**B**) identified nodes representing the analysed systemic photosensitising drugs among the 25 author-keyword nodes meeting the minimum occurrence threshold. Because the co-occurrence analysis included only author keywords occurring at least four times, the absence of these drug-related nodes was interpreted as a feature of the thematic structure of the mapped corpus rather than as evidence of an absolute absence of relevant literature.

The generated co-occurrence network of author keywords (comprising 25 nodes with a minimum threshold of 4 occurrences) revealed a distinct partition of the existing literature into five coherent thematic clusters (Figure 1). The first cluster (Cluster 1), centered on classical photodynamics and reactive oxygen species generation, represents the traditional approach to photosensitivity through terms such as photosensitizer, singlet oxygen, photodynamic therapy, methylene blue, and riboflavin. The second cluster (Cluster 2) pertains to localized microbial photoinactivation in clinical settings, linking photodynamic inactivation and phototoxicity with diode laser applications and indocyanine green, predominantly within endodontic infection contexts. The third thematic domain (Cluster 3) encompasses nanomedicine and materials engineering, focusing on advanced drug delivery systems and light-converting nanostructures, characterized by keywords such as upconversion, drug delivery, and self-assembly. The fourth cluster (Cluster 4) addresses diagnostic methodologies and optical biophysics leveraging near-infrared properties, including near-infrared, fluorescence, and optogenetics. Finally, the fifth cluster (Cluster 5) aggregates studies on photothermal phenomena alongside tissue regeneration and biostimulation, exemplified by photothermal therapy, photobiomodulation, and wound healing.

The Scopus corpus was sequentially refined using the “Search within results” function. The first query (fibroblast OR dermal OR “aesthetic medicine” OR ketoprofen OR meloxicam OR hydrochlorothiazide OR doxycycline OR diclofenac) reduced the dataset from 391 to 49 records. Because the terms were combined using the OR operator, this step identified records containing at least one term related either to the dermal/aesthetic context or to one of the investigated drugs. A second query restricted to ketoprofen OR meloxicam OR hydrochlorothiazide OR doxycycline OR diclofenac reduced the dataset from 49 to 9 records. These steps represented database-based refinement rather than manual title/abstract screening. Subsequent assessment of the nine identified publications showed that none directly evaluated the effects of 980 nm diode laser irradiation on human dermal fibroblasts in the presence of ketoprofen, meloxicam, hydrochlorothiazide, doxycycline, or diclofenac.

The nine publications identified after the final refinement step are summarized in Table S1.

### 3.2. Cell Viability Across All Experimental Conditions

**Figure 2:**
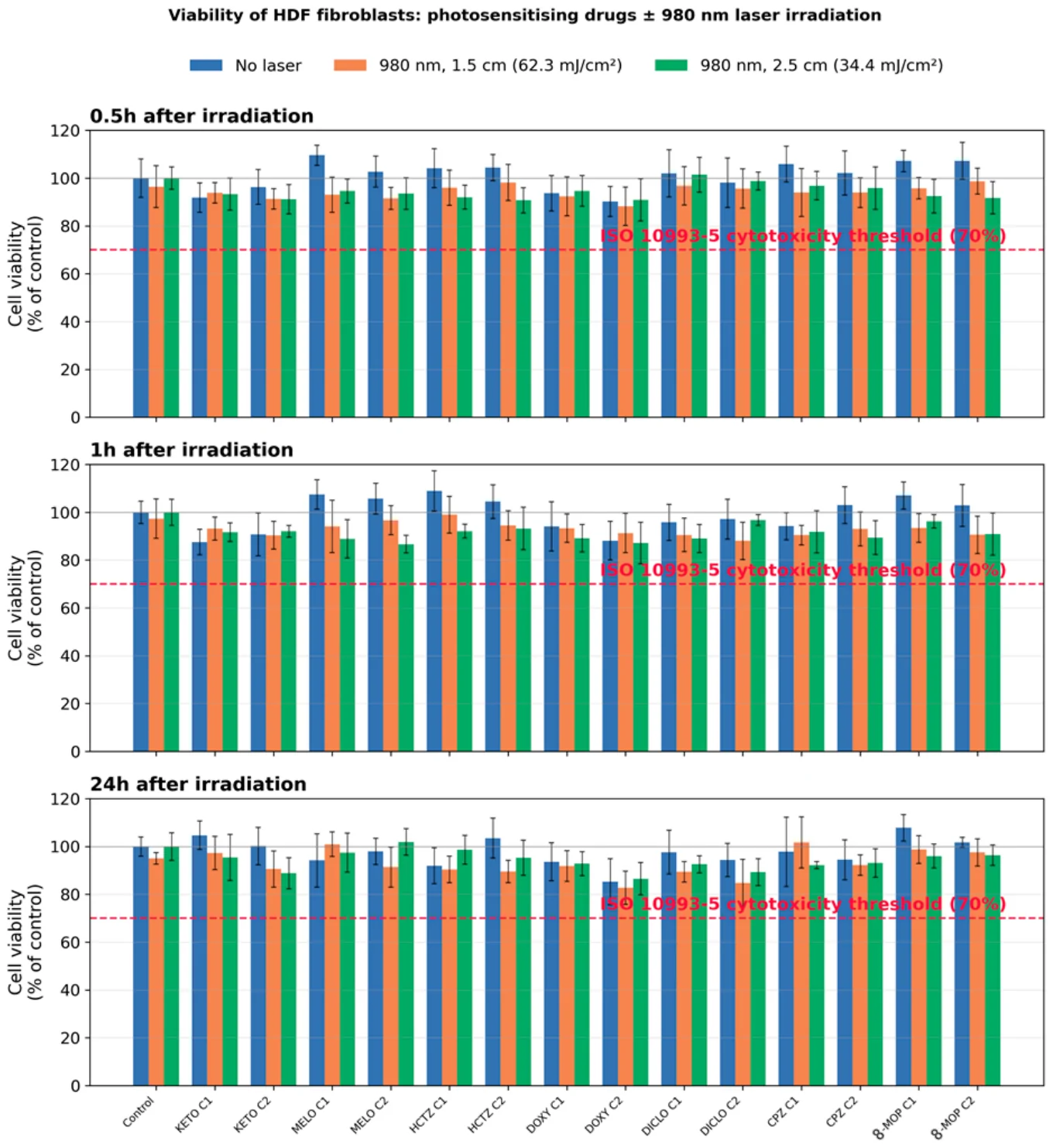
Viability of normal human dermal fibroblasts (HDFs) exposed to seven photosensitising drugs, each at two concentrations (C1, C2), in the absence of irradiation and after 980 nm diode-laser irradiation at two source-to-plate distances (1.5 cm, 62.3 mJ/cm^2^; 2.5 cm, 34.4 mJ/cm^2^). Viability is expressed as a percentage of the untreated control (mean ± SD; n = 3), and reported separately for 0.5, 1 and 24 h after irradiation (upper, middle and lower panel). The horizontal dashed line marks the ISO 10993-5 cytotoxicity threshold (70%); the solid grey line indicates the control level (100%). KETO, ketoprofen; MELO, meloxicam; HCTZ, hydrochlorothiazide; DOXY, doxycycline; DICLO, diclofenac; CPZ, chlorpromazine; 8-MOP, 8-methoxypsoralen.

Cell viability was quantified for 15 experimental groups (untreated control and seven photosensitising drugs, each at two concentrations), under three irradiation conditions (no laser; 980 nm at 1.5 cm, 62.3 mJ/cm^2^; 980 nm at 2.5 cm, 34.4 mJ/cm^2^) and at three post-irradiation time points (0.5, 1 and 24 h), giving 135 mean values in total (Figure 2). Across the entire matrix, viability remained high, ranging from 82.7% to 109.6% of the untreated control. Crucially, no condition—drug alone, laser alone, or drug combined with 980 nm irradiation—reduced mean viability below the ISO 10993-5 cytotoxicity threshold of 70% [22] (dashed line in Figure 2), so by the standard regulatory criterion, none of the tested treatments was cytotoxic to normal human dermal fibroblasts.

The drug-only (dark) conditions confirmed that the compounds themselves were well tolerated at the concentrations used: viability in non-irradiated, drug-treated wells ranged from 85.3% (doxycycline, higher concentration, 24 h) to 109.6% (meloxicam, lower concentration, 0.5 h). Notably, the two classical photosensitisers, chlorpromazine and 8-methoxypsoralen—both of which are strongly phototoxic under ultraviolet A—did not reduce viability under 980 nm irradiation, with values remaining between 90% and 108% of control throughout. This is consistent with the wavelength-specificity of their photoreactivity and indicates that the near-infrared exposure did not activate even these highly photolabile molecules.

### 3.3. Spectral Stability Analysis in the UV–Vis Region

**Figure 3:**
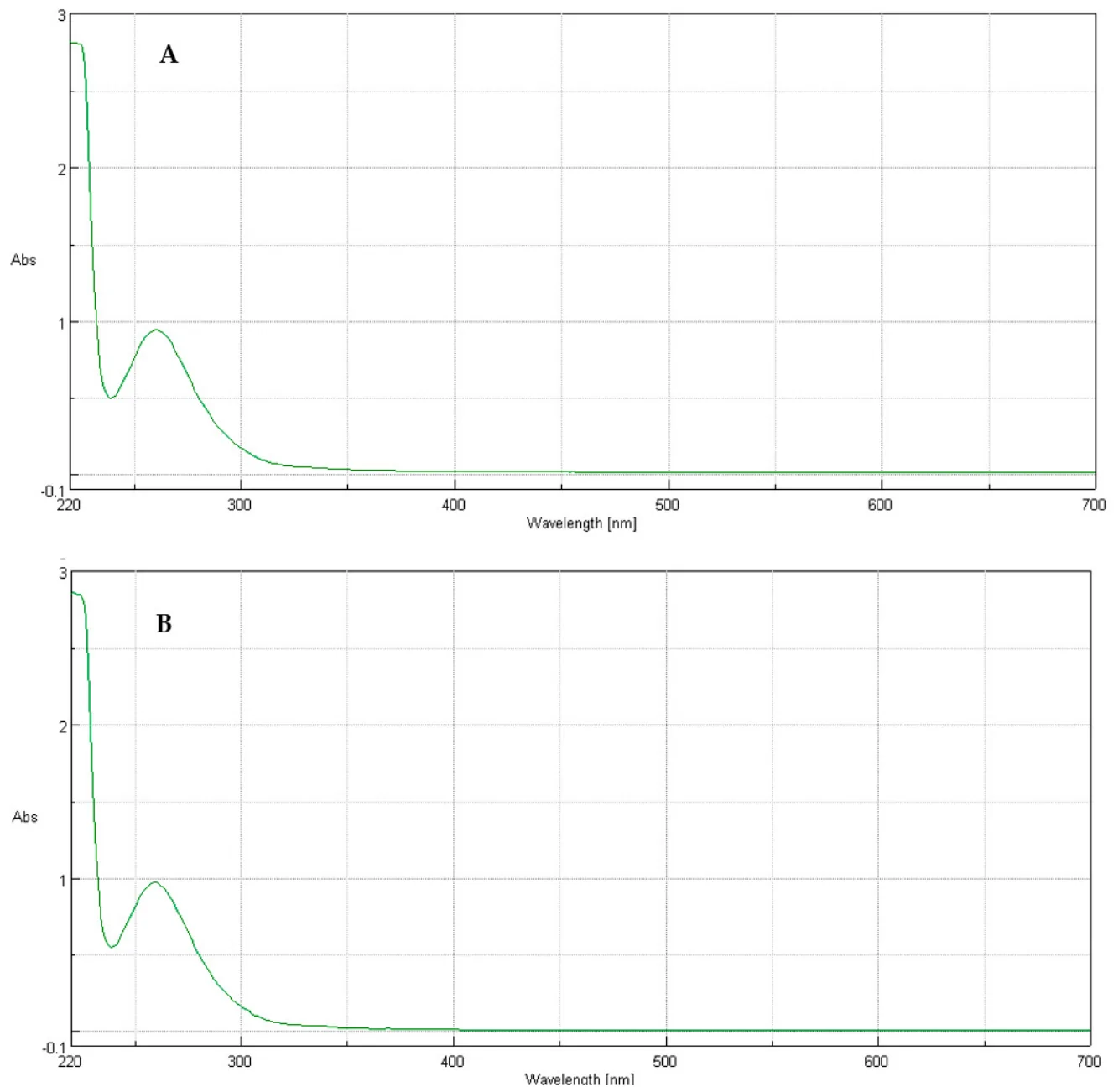
UV–Vis absorption spectra of 50 µM ketoprofen recorded before and after laser irradiation. (**A**) Absorption spectrum of the non-irradiated ketoprofen solution (control). (**B**) Absorption spectrum of the ketoprofen solution following laser irradiation. No significant changes in the spectral profile, absorbance at λ_max (260 nm), or the appearance of additional absorption bands were observed, indicating that laser exposure did not induce detectable degradation of ketoprofen under the applied experimental conditions.

The UV–Vis spectrophotometric analysis demonstrated no evidence of the drug degradation under the applied experimental conditions. Comparison of the absorption spectra recorded before and after laser irradiation revealed no significant differences in the spectral profiles (Figure 3). The absorbance values at the characteristic absorption maximum (λ_max = 260 nm) remained within a narrow range of 0.96–0.99 for both irradiated and non-irradiated samples, indicating that laser exposure did not affect the optical properties of ketoprofen. Furthermore, no additional absorption bands or spectral shifts were observed following irradiation, suggesting the absence of detectable photodegradation products or structural alterations. These findings confirm that the applied laser irradiation conditions did not compromise the chemical stability of the investigated drug.

### 3.4. The Drugs Did Not Potentiate Light-Induced Injury (Compound × Laser Interaction)

To separate any general effect of irradiation from an effect specific to the drugs, a two-way analysis of variance (factor A = compound/concentration, 15 levels; factor B = laser condition, 3 levels) was performed for each time point. The compound × laser interaction was non-significant at every time point (F = 0.84, *p* = 0.69 at 0.5 h; F = 1.21, *p* = 0.25 at 1 h; F = 0.89, *p* = 0.62 at 24 h). Because a drug-dependent amplification of light-induced damage is the defining feature of phototoxicity, the absence of this interaction is the central result of the study: the presence of a photosensitising drug did not change how the fibroblasts responded to 980 nm irradiation.

This conclusion was corroborated at the level of individual comparisons. None of the 90 pairwise contrasts between an irradiated condition and its matched non-irradiated counterpart remained statistically significant after Benjamini–Hochberg correction for multiple testing (all adjusted *p* ≥ 0.47). The largest single nominal reduction attributable to irradiation (meloxicam, higher concentration, 2.5 cm, 1 h: −19.1 percentage points; uncorrected *p* = 0.018) lost significance after correction and was recorded at the lower of the two fluences—the opposite of what genuine dose-dependent photodamage would predict.

### 3.5. A Minor, Drug- and Fluence-Independent Effect of Irradiation

**Figure 4:**
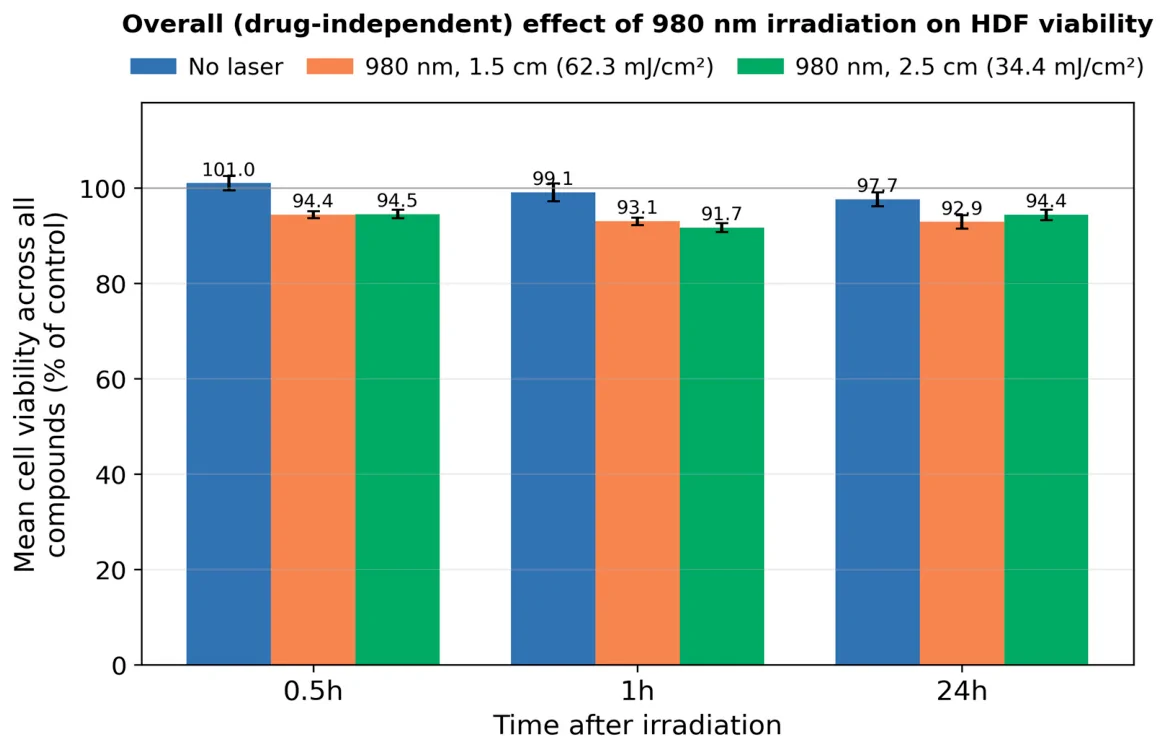
Overall drug-independent effect of 980 nm irradiation on HDF viability. Mean cell viability averaged across all 15 experimental groups (±SEM) is shown for each irradiation setting (no laser; 1.5 cm; 2.5 cm) at 0.5, 1 and 24 h after exposure. The reduction of approximately six percentage points under irradiation was independent of the applied fluence (near-identical values at 62.3 and 34.4 mJ/cm^2^) and remained well above the ISO 10993-5 cytotoxicity threshold, arguing against a dose-dependent phototoxic mechanism.

The two-way ANOVA did detect a small but statistically significant main effect of irradiation (factor B) at all time points (F = 13.3, *p* < 10^−5^ at 0.5 h; F = 14.7, *p* < 10^−5^ at 1 h; F = 5.4, *p* = 0.006 at 24 h). Averaged across all compounds, viability was 99.3% without laser and 93.5% under irradiation, i.e., a mean reduction of approximately six percentage points (Figure 4). Three independent lines of evidence show that this modest effect does not constitute phototoxicity.

First, the reduction was present to the same degree in the drug-free control wells (control viability of 95–97% under irradiation versus 100% without laser), demonstrating that it is not mediated by the drugs. Second, it was essentially identical at the two fluences (93.5% at 62.3 mJ/cm^2^ and 93.5% at 34.4 mJ/cm^2^; Figure 4), i.e., it did not scale with radiant exposure, whereas true photochemical or photothermal injury would be expected to increase with fluence. Third, the effect was small in absolute terms and remained far above the cytotoxicity threshold at every time point. Together these observations indicate that the six-percentage-point decrease reflects a mild, non-specific consequence of the irradiation and handling procedure rather than photochemically driven, drug-mediated injury.

### 3.6. Non-Cytotoxicity and Equivalence Assessments

In the equivalence framework, every group retained a mean viability above the 70% cytotoxicity threshold, and point estimates lay close to the control across the full data set. Because the study was based on a limited number of replicates, the 95% confidence intervals were relatively wide (the lowest interval reaching approximately 60%), so strict statistical equivalence within the pre-specified ±20-percentage-point margin could not be formally declared for every individual comparison. This is a limitation of statistical power rather than an indication of harm: the observed effects were consistently small and non-cytotoxic. Confirmation with a larger number of independent replicates is therefore recommended to establish formal equivalence, and this is addressed in the Discussion.

### 3.7. Behaviour of the Individual Drug Classes

The non-steroidal anti-inflammatory drugs of principal interest for aesthetic practice—ketoprofen, diclofenac and meloxicam—did not reduce fibroblast viability below the non-cytotoxic range under 980 nm irradiation at either fluence or at any time point, and showed no evidence of a drug-specific interaction with the laser. The tetracycline doxycycline produced the lowest viabilities of the series at its higher concentration (as low as 82.7%), but this mild reduction was already present in the non-irradiated wells and did not increase with irradiation, identifying it as a modest intrinsic (dark) effect of the drug rather than a phototoxic response. The thiazide hydrochlorothiazide and both positive-control photosensitisers behaved similarly, with viabilities remaining within the non-cytotoxic range under all irradiation conditions.

### 3.8. Summary of Findings

In summary, 980 nm laser irradiation of normal human dermal fibroblasts, whether applied alone or in the presence of seven established photosensitising drugs, did not produce cytotoxicity, and the drugs did not potentiate any light-induced loss of viability (Figure 2 and Figure 3). The only measurable effect of irradiation was a minor, drug-independent and fluence-independent decrease in viability that remained far above the cytotoxicity threshold.

## 4. Discussion

The bibliometric analysis delineated distinct thematic domains within the retrieved Scopus corpus, including NIR biostimulation, photothermal therapy, and photodynamic antimicrobial inactivation. However, within the corpus retrieved using the predefined search strategy and subsequent refinement steps, no experimental study was identified that directly evaluated 980 nm diode laser irradiation of human dermal fibroblasts in the presence of ketoprofen, meloxicam, hydrochlorothiazide, doxycycline, or diclofenac. The present experimental study addresses this specifically defined evidence gap by directly evaluating fibroblast viability under these conditions.

The present study was designed to test a mechanistically motivated hypothesis: that the phototoxic potential attributed to a range of commonly used drugs—a property established almost exclusively from ultraviolet A (UVA) exposure—does not necessarily extend to the near-infrared (NIR) wavelengths at which many contemporary medical and aesthetic lasers operate [1,2]. Using normal human dermal fibroblasts irradiated with a 980 nm diode laser, we observed no drug-specific phototoxicity and no cytotoxicity for any of the seven photosensitisers examined, including the archetypal UVA photosensitisers chlorpromazine and 8-methoxypsoralen. Viability remained above the ISO 10993-5 cytotoxicity threshold of 70% [22] under every condition, and the compound × laser interaction—the statistical signature of drug-potentiated photodamage—was absent at all time points. These findings provide direct experimental support for the view that the ultraviolet-defined phototoxicity of these agents is wavelength-specific and is not reproduced at 980 nm.

### 4.1. Mechanistic Interpretation: The Primacy of the Action Spectrum

The result is coherent with the fundamental photophysics of drug phototoxicity. A photochemical reaction can only be initiated at wavelengths absorbed by the drug (or its photoproducts); absorption populates excited singlet and triplet states from which type I (radical) and type II (singlet-oxygen) pathways generate the reactive species responsible for lipid, protein and DNA damage [1,3]. For the compounds studied here, this absorption lies in the UVA region: the benzophenone chromophore of ketoprofen, the oxicam system of meloxicam, the tetracycline scaffold of doxycycline and the furocoumarin nucleus of 8-methoxypsoralen all absorb UV rather than NIR light [1,6,7,14]. At 980 nm, these chromophores are essentially not excited, so the excited-state photochemistry that underlies phototoxicity cannot proceed. The most persuasive internal evidence for this interpretation is the behaviour of the reference photosensitisers chlorpromazine and 8-methoxypsoralen, which are potent phototoxic and photogenotoxic agents under UVA and in psoralen-UVA (PUVA) photochemotherapy [14], were entirely inert in our NIR paradigm. That drugs known to be strongly phototoxic in the very same classes of skin cells under UVA [7,9,10] produced no measurable injury at 980 nm underscores that wavelength, not merely the presence of a “photosensitiser”, governs the outcome. It is precisely for this reason that the validated regulatory assay for photoreactivity restricts irradiation to the UVA/visible region [15].

### 4.2. Interpreting the Minor, Non-Specific Effect of Near-Infrared Exposure

The small, drug-independent and fluence-independent reduction in viability (~6 percentage points) is best understood within the framework of NIR photobiology rather than photochemistry. Water is a principal chromophore at 980 nm, and part of the incident energy is dissipated as heat; moreover, NIR–tissue interactions characteristically follow a biphasic (Arndt-Schulz) dose–response, in which exposure can be stimulatory, neutral or mildly inhibitory depending on parameters [21,23]. The two observations that this effect was equally present in drug-free controls and did not scale with fluence are difficult to reconcile with a photochemical, drug-mediated mechanism and instead point to a mild, non-specific influence of the irradiation and handling procedure [18,23]. This is consistent with the broader photobiomodulation literature in which 980 nm and other NIR sources modulate fibroblast viability and metabolism in a generally reversible, context-dependent manner [19,20]. Importantly, in the present data this modulation never approached the cytotoxic range, reinforcing that it represents a benign physiological response rather than damage.

### 4.3. Relationship to the Existing Literature and Assay Sensitivity

Our conclusions should be read against the substantial body of evidence documenting the UVA phototoxicity of these same drugs. Doxycycline is the most phototoxic tetracycline, with an action spectrum dominated by long-wave UVA [11,12]; hydrochlorothiazide has a UVA-driven photosensitising and possible photocarcinogenic profile that has prompted controlled photobiological study [13]; and diclofenac phototoxicity proceeds through UVA-generated photoproducts and reactive oxygen species [8]. The contrast between those studies and our NIR-null result is therefore not a contradiction but a demonstration of wavelength dependence. It is also relevant that our assay was not blind to real toxicity: doxycycline at the higher concentration produced the lowest viabilities of the series, yet this reduction was already present without irradiation and was not increased by the laser, identifying it as a modest intrinsic (dark) drug effect rather than a phototoxic one. This internal sensitivity to a genuine, non-photic effect strengthens confidence in the idea that the absence of a NIR phototoxic signal reflects biology rather than an insensitive readout.

### 4.4. Implications for Aesthetic and Laser Medicine

These findings may be relevant to future clinical risk assessment, as the use of photosensitising medications is frequently regarded as a relative contraindication to laser procedures [2]. Clinical protocols using 980 nm diode lasers vary considerably according to the indication, device characteristics and mode of energy delivery. Desiate et al. reported treatment of vascular lesions using pulse durations of 10–50 ms, a 2 mm spot diameter and fluences of 6–10 J/cm^2^, whereas Wollina described transcutaneous treatment of benign vascular skin lesions using powers of 25–40 W and pulse durations of 10 ms or less [16,17]. These reported clinical parameters are higher than those applied in the present in vitro model, indicating that further studies are needed to determine whether similar results would be observed under higher-exposure conditions. In the present study, none of the investigated photosensitising drugs enhanced the reduction in fibroblast viability induced by 980 nm irradiation. These findings suggest that phototoxicity established for ultraviolet exposure should not automatically be assumed to occur at near-infrared wavelengths. At the same time, the observations are specific to 980 nm irradiation and should not be directly extended to optical systems emitting in the visible or ultraviolet range, including broadband devices such as IPL, whose emission spectra may overlap the absorption bands of photosensitising compounds. Drug-associated photosafety should therefore be evaluated in relation to the spectral characteristics and exposure conditions of the individual optical modality.

### 4.5. Photosensitising Drugs and Photoprotection: A Wavelength-Specific Rationale

It is essential that these results are not misconstrued as diminishing the importance of photoprotection. Patients taking photosensitising drugs remain at genuine risk from solar and artificial UVA exposure, and rigorous broad-spectrum photoprotection—sunscreens with strong UVA coverage, sun avoidance and protective clothing—continues to be an indispensable part of their care [2,5,24]. The conceptual contribution of our study is to clarify why: the risk is concentrated in the ultraviolet region of the spectrum, where these drugs absorb and where their photochemistry is triggered. The same mechanistic rationale that mandates vigorous UVA-directed photoprotection is also consistent with the absence of detectable phototoxicity under the NIR exposure conditions tested here. In this sense, our findings do not relax the case for photoprotection but sharpen it: sunscreens and UVA filters remain important for these patients [24], and framing their necessity in terms of the drug action spectrum both reinforces adherence for solar exposure and rationalises a more measured approach to NIR laser procedures. Patients taking photosensitising medications should continue to receive appropriate counselling regarding rigorous UVA photoprotection. In our study, no detectable phototoxic effect was observed under the NIR exposure conditions tested. However, these findings are limited to the in vitro model used and require further confirmation before they can be translated into clinical recommendations for NIR-based aesthetic procedures.

### 4.6. Broader Significance and Future Directions

More generally, these results argue for an action-spectrum-based, wavelength-resolved approach to communicating drug photosafety, in place of an undifferentiated “photosensitising” label applied across all light sources [1,2]. Future work should extend the present observations with adequately powered equivalence designs, additional and more sensitive endpoints (intracellular reactive oxygen species, DNA strand breaks and apoptotic markers), a wider range of skin cell types and reconstructed three-dimensional skin models, and, ultimately, in vivo confirmation. Systematic mapping of drug phototoxicity across the ultraviolet, visible and near-infrared regions would provide the evidence base needed for precise, device-specific clinical guidance.

### 4.7. Study Limitations

Several limitations temper the interpretation of these findings. First, this is an in vitro study performed on a fibroblast monolayer; although fibroblasts are a relevant target of dermally penetrating NIR light, they do not capture the responses of keratinocytes, melanocytes, endothelial or immune cells, nor the architecture of reconstructed three-dimensional skin or intact tissue in vivo. Second, the optical exposure investigated in this study was intentionally limited to a single wavelength (980 nm), one pulsed irradiation regimen and two low fluences selected on the basis of preliminary experiments to minimise the intrinsic cytotoxic effect of laser irradiation. Consequently, the results cannot be generalised to higher fluences, continuous-wave irradiation, or other devices emitting near-infrared, visible or broadband light, including intense pulsed light (IPL) systems. Third, the endpoint was cell viability, which reports metabolic activity and membrane integrity but not sublethal injury; oxidative stress, DNA damage, apoptosis and delayed or proliferative effects were not assessed and warrant dedicated assays (for example intracellular ROS quantification and the comet assay). Fourth, the analysis was based on a limited number of replicates, which widened the confidence intervals and left the study underpowered for a formal, categorical demonstration of equivalence within the pre-specified margin; larger, independent replication is required to establish equivalence definitively. Fifth, in vitro drug concentrations and exposure timing may not reproduce the dermal pharmacokinetics, distribution and photoproduct formation that occur in vivo. Finally, sample temperature during irradiation was not continuously monitored, so a minor thermal contribution to the small, non-specific effect described above cannot be formally excluded. These limitations define a clear agenda for confirmatory studies but do not undermine the central, mechanistically grounded observation that the drugs tested did not exert phototoxicity toward normal fibroblasts under 980 nm laser irradiation. As the present investigation represents a preliminary screening study, further mechanistic studies are warranted. In particular, measurements of intracellular ROS and DNA damage should be prioritized to identify sublethal photochemical effects that may not result in detectable changes in WST-1 activity. Subsequent validation using 3D reconstructed human skin models would also provide a more physiologically relevant assessment of potential phototoxic responses and improve the translational relevance of the findings.

## 5. Conclusions

This study demonstrated that irradiation of normal human dermal fibroblasts with a 980 nm diode laser (62.3 and 34.4 mJ/cm^2^) did not induce cytotoxicity, and that none of the seven established photosensitising drugs examined—ketoprofen, meloxicam, hydrochlorothiazide, doxycycline, diclofenac, and the reference photosensitisers chlorpromazine and 8-methoxypsoralen—potentiated any light-induced loss of viability. Cell viability remained above the ISO 10993-5 cytotoxicity threshold under every condition, and, decisively, no compound × laser interaction was detected, indicating the absence of drug-specific phototoxicity at this near-infrared wavelength. The only measurable effect of irradiation was a minor, drug- and fluence-independent reduction in viability that stayed well within the non-cytotoxic range and is most consistent with a benign, non-photochemical response.

Taken together, these results support the central hypothesis of the work: the phototoxicity of these drugs, which is defined by their absorption in the ultraviolet A region, does not extend to 980 nm near-infrared laser light. These findings suggest that the need for automatic exclusion of patients receiving photosensitising medications from near-infrared diode-laser procedures may warrant re-evaluation, although clinical studies under relevant treatment conditions are required before any change in practice can be recommended. At the same time, this reassurance is wavelength-specific and does not diminish the importance of photoprotection: for these patients, rigorous broad-spectrum, UVA-directed photoprotection with sunscreens and filters remains essential to protect against solar and ultraviolet exposure because that is precisely where their drug-related risk resides.

These conclusions are limited to the specific in vitro conditions tested and should not be extrapolated to higher fluences, continuous-wave NIR irradiation, or broadband light sources such as IPL. Further studies using additional cellular endpoints, skin models, irradiation parameters, and ultimately in vivo settings are required before the findings can be translated into clinical practice. Moreover, a limitation of the present study is that a dedicated assay positive control with a well-established phototoxic response under 980 nm irradiation was not included. Therefore, the present findings should be interpreted as a comparative assessment of drug- and irradiation-dependent changes in cell viability under the experimental conditions applied, rather than as a formal validation of the assay for phototoxicity testing at 980 nm. Nonetheless, the findings provide a mechanistically coherent, quantitative basis for a more nuanced, wavelength-resolved assessment of drug photosafety and for safer, better-informed use of near-infrared lasers in patients taking photosensitising medications.

## Data Availability

Data is contained within the article or Supplementary Materials. The data presented in this study are available on request from the corresponding author.

## Supplementary Materials

The following supporting information can be downloaded at: https://www.mdpi.com/article/10.3390/pharmaceutics18101259/s1, Table S1: Publications identified after the final Scopus refinement step [27,28,29,30,31,32,33,34,35].

## Abbreviations

The following abbreviations are used in this manuscript:

DMSO	Dimethyl sulfoxide
HDFs	Human dermal fibroblasts
IPL	Intense pulsed light
NIR	Near-infrared
PDT	Photodynamic therapy
PUVA	Psoralen plus ultraviolet A
ROS	Reactive oxygen species
UV	Ultraviolet
UVA	Ultraviolet A
UVB	Ultraviolet B
