# Supplementary material for: Evaluation of Drug-Associated Phototoxicity Under 980 nm Near-Infrared Laser Irradiation in Human Dermal Fibroblasts
Source: Pharmaceutics. 2026 Oct 6;18(10):1259. doi: 10.3390/pharmaceutics18101259 (PMC13708388; doi:10.3390/pharmaceutics18101259)
Supplement: Supplementary file 1 [file pharmaceutics-18-01259-s001.zip › pharmaceutics-4557112-supplementary.pdf]

Supplementary Table S1

Publications identified after the final Scopus refinement step.

	Publication	Document Type
1.	Permatasari, P.A.D.; Astuti, S.D.; Yaqubi, A.K.; Paisei, E.A.W.; Pujiyanto; Anuar, N. Effectiveness of Katuk Leaf Chlorophyll (Sauropus Androgynus (L) Merr) with Blue and Red Laser Activation to Reduce Aggregatibacter Actinomycetemcomitans and Enterococcus Faecalis Biofilm. *Biomedical Photonics* 2023, *12*, 14–21. doi:10.24931/2413-9432-2023-12-1-14-21 [27].	Article
2.	Hou, Y.; Meng, X.; Zhang, S.; Sun, F.; Liu, W. Near-Infrared Triggered Ropivacaine Liposomal Gel for Adjustable and Prolonged Local Anaesthesia. *International Journal of Pharmaceutics* 2022, *611*, 121315, doi:10.1016/j.ijpharm.2021.121315 [28].	Article
3.	Garini, S. W., Astuti, S. D., Kusumawati, I., & Susilo, Y. (2021). Combination of curcumin photosensitizer with laser diode to reduce antibiotic resistant bacterial biofilms. *Malaysian Journal of Medicine and Health Sciences*, *17*, 78-81 [29].	Article
4.	Hu, X.; Zhao, H.; Liang, Y.; Chen, F.; Li, J.; Chen, R. Broad-Spectrum Response NCQDs/Bi2O2CO3 Heterojunction Nanosheets for Ciprofloxacin Photodegradation: Unraveling the Unique Roles of NCQDs upon Different Light Irradiation. *Chemosphere* 2021, *264*, 128434, doi:10.1016/j.chemosphere.2020.128434 [30].	Article
5.	Zaleski-Larsen, L.A.; Fabi, S.G. Laser-Assisted Drug Delivery. *Dermatologic Surgery* 2016, *42*, 919–931. doi:10.1097/DSS.0000000000000556 [31].	Review
6.	Heymann, P.G.B.; Mandic, R.; Kämmerer, P.W.; Kretschmer, F.; Saydali, A.; Neff, A.; Draenert, F.G. Laser-Enhanced Cytotoxicity of Zoledronic Acid and Cisplatin on Primary Human Fibroblasts and Head and Neck Squamous Cell Carcinoma Cell Line UM-SCC-3. *Journal of Cranio-Maxillofacial Surgery* 2014, *42*, 1469–1474, doi:10.1016/j.jcms.2014.04.014 [32].	Article
7.	Nardi, G.; Manet, I.; Monti, S.; Miranda, M.A.; Lhiaubet-Vallet, V. Scope and Limitations of the TEMPO/EPR Method for Singlet Oxygen Detection: The Misleading Role of Electron Transfer. *Free Radical Biology and Medicine* 2014, *77*, 64–70, doi:10.1016/j.freeradbiomed.2014.08.020 [33].	Article
8.	El Yazami, H.; Zeinoun, T.; Bou Saba, S.; Lamard, L.; Peremans, A.; Limme, M.; Geerts, S.; Lamy, M.; Nammour, S. Pulp Temperature Increase during Photo-Activated Disinfection (PAD) of Periodontal Pockets: An in Vitro Study. *Lasers in Medical Science* 2010, *25*, 655–659, doi:10.1007/s10103-009-0686-z [34].	Article
9.	de la Peña, D.; Martí, C.; Nonell, S.; Martínez, L.A.; Miranda, M.A. Time-Resolved Near Infrared Studies on Singlet Oxygen Production by the Photosensitizing 2-Arylpropionic Acids. *Photochemistry and Photobiology* 1997, 65, 828–832, doi:10.1111/j.1751-1097.1997.tb01930.x [35].	Article
